# Does Proof of Concept Trump All? RRI Dilemmas in Research Practices

**DOI:** 10.1007/s11948-021-00288-8

**Published:** 2021-02-02

**Authors:** Anita Borch, Harald Throne-Holst

**Affiliations:** Consumption Research Norway (SIFO), OsloMet – Oslo Metropolitan University, Stensberggata 26, N-0130 Oslo, Norway

## Abstract

Responsible Research and Innovation (RRI) is described as a new way of doing science that brings science closer to society. Based on a qualitatively oriented case study, this article supports previous research indicating that researchers face a variety of ethical problems and dilemmas when implementing RRI for the first time. These include difficulties with anticipating and controlling future impacts, an asymmetry of power between project partners and an elusive understanding of the RRI concept. The researchers’ challenges were rooted in conventional research ethics and could be boiled down to one core dilemma: If RRI had been applied from the very beginning of the project period, the chance of realising proof of concept within the scheduled time may decrease. The researchers’ solution to this dilemma was to prioritize proof of concept and postpone RRI activities to later stages of the project. If RRI is expected to live up to its ambition of representing a new way of doing science, more effort is needed at the political level to facilitate change.

## Introduction

Over the last decades, the dominant ways of conducting science have been challenged by *responsible research and innovation* (RRI) (Stahl et al. 2019). RRI is outlined in the Rome declaration as ‘the on-going process of aligning research and innovation to the values, needs, and expectations of society’ (European Commission 2014). In RRI, societal actors work together during the whole Research and Innovation (R&I) process in order to better align R&I outcomes to commonly (European) shared values. The matter of what we, as a society, want out of science and technology is as, if not more important, than, say, health, safety and risk management.

Since 2009, the political and academic discourse on RRI has experienced exceptional growth (Timmermans 2017). Although the strength of the discourse has stabilised or decreased over the last few years (Silva et al. 2019), its emphasis on ‘societal and environmental impact’ and ‘user involvement’ are still pursued in important research calls such as the new Horizon Europe Framework Programme for 2020 to 2027 (Von Schomberg and Hankins 2019). So far, most research on RRI has been conceptual, addressing frameworks and approaches, whereas less attention has been paid to practical issues relating to its implementation such as challenges, dilemmas and constraints (Nathan 2015; Timmermans 2017; Kuzma and Roberts 2018; Ribeiro et al. 2018). To address this knowledge gap and to better understand potential barriers to the widespread adoption of RRI, this paper focuses on the challenges faced by researchers who are meeting the RRI framework for the first time. This focus is warranted on the basis of two primary observations; firstly, because we have found these challenges to be most explicitly articulated in the first phase of an R&I processes, and secondly, because the research results presented in this paper will speak directly to those most in need of this kind of knowledge, i.e. researchers who are about to implement mandatory RRI for the first time.

One of the barriers most frequently described in the RRI literature is that researchers have no clear idea of what the RRI process might look like, how it may impact their daily experiences, and at what cost (Stahl et al. 2013; Blok and Lemmens 2015; Burget et al. 2017).

Another frequently discussed challenge is known as the ‘Collingridge dilemma’. This refers to a double-bind problem (Collingridge 1980: 1) that impacts cannot be easily anticipated until the technology has been extensively developed and widely used (information problem) and 2) that control or change is difficult when the technology has become entrenched (a power problem). Collingridge’s suggestion to solve this dilemma was to ensure that decisions made during R&I processes were reversible, corrigible and flexible. The challenges associated with identifying ethical issues in advance have been discussed by several scholars. At one extreme,some have argued that there is no way out of the Collingridge dilemma: ‘Instead, we have to seek to intervene under conditions of uncertainty and armed with little more than good will.’(Nordmann (2018, 336)). At the other extreme, others have indicated that the Collingridge dilemma is no obstacle to practising RRI: ‘[From a] communication-oriented view of ethics, it is not necessarily assumed that ethical issues can be identified in advance … Instead, an open exchange between researchers and stakeholders is employed to identify and highlight possible issues’ (Stahl et al. 2019: 5).

A third problem that may hinder change in the ways in which science is widely assumed to work regards the different actors involved in R&I processes and the relationship between them. Not only do relevant actors involved in R&I processes represent different interests and values (Millar et al. 2012; de Bakker et al. 2014; Nathan 2015; Ribeiro et al. 2018; Silva et al. 2019), but there also appears to be an asymmetry of information, competence and power between them. Based on a literature review, Silva et al. (2019) found that the involvement of stakeholders in RRI tends to be initiated by academic researchers, for the most part takes place when the innovation has already reached the market, and involves limited product and paradigm innovations. Whereas members of consortia are truly involved in R&I processes, stakeholders (e.g. governments, competitors, consumer advocates, environmentalists, special interest groups and the media) tend to play more of an advisory role. An asymmetry is also found between members of consortia, for example between scientists and social scientists, the latter reflecting that the SSHs, the ‘soft sciences’, have often found themselves at a disadvantage compared with the ‘hard’ sciences and engineering (Storer 1967), which makes interdisciplinary cooperation and collaboration more challenging (Felt 2014; Levidow and Neubauer 2014).

From this literature review, we can conclude that researchers do experience barriers and ethical dilemmas when implementing RRI. To some extent, we also get an idea of what these barriers are. Very little is, however, known about how dilemmas are handled by researchers. As the researchers’ handling of dilemmas may not only promote but also hinder widespread adoption RRI, more knowledge on this subject is needed.

The research questions of this paper are as follows:

- What dilemmas do researchers face when practising mandatory RRI for the first time?
- How do scientists and the SSH scholars solve these dilemmas?
- How do these solutions affect the change from conventional research practices to RRI practices?
- If desirable, how can conventional research practices be dissolved and replaced by RRI practices?

Dilemmas are here understood as situations where researchers are forced to choose one moral principle at the expense of another. Such situations are most likely more frequent in RRI practices than in conventional research practices, as the former involve more stakeholders with different interests and priorities (Nathan 2015, 2010). Although ethical dilemmas are experienced by individuals, they are closely connected to the cultural, economic, institutional and political environment of which they form part, and are articulated and materialised/digitalised, for example, in the form of expectations formulated in conventional research (ethical) principles and RRI goals and dimensions enshrined in textbooks and project applications. In this respect, ethical dilemmas experienced at the individual level are also a social responsibility (Forsberg et al. 2018).

Research practices are here understood as routinised types of behaviour involving the performance of, and integration between, three elements: understandings of what the practices are and how they should be performed, competences to carry out the practices, and the materials (resources, labs, lab equipment, textbooks etc.) required to do so (Borch et al. 2015). These three elements are inspired by practice theoretical approaches (here derived from the definition proposed by Reckwitz (2002). These have gained ground in the social sciences in recent years since they, in contrast to most social theory, place more emphasis on what people do than on what they say, and highlight the role of materials (things, technologies, physical surroundings and infrastructures) in social life (Nicolini 2012).

The paper starts with a section outlining the theories and methodologies on which the analysis is based, followed by two sections that analyse and discuss the main findings.

### Conventional research ethics versus RRI

Up until World War II science tended to be seen as something that was inherently ‘good’ (cf. Vannevar Bush’s report ‘Science – The endless frontier’ from 1945). The purpose of science was to deliver new and useful science to society, and to achieve this, science and its institutions had to be assured significant independence and freedom. Today, most researchers are trained within a scientific paradigm where prevailing ideas of what science is and how it shall be conducted are most explicitly articulated in what we in this article will call ‘conventional research ethics’ (CRE). In the context of this paper, CRE refers to research ethical principles or guidelines that are often standardised, formalised and ideally institutionalised and practiced across disciplinary, national and cultural boundaries. They are typically developed and undertaken by international review boards (IRBs) or research ethical committees (RECs) (Stahl et al. 2019). Like most rules, CREs are not necessarily implemented in practice, for example due to people having different opinions of ethical standards and therefore may to adopt their own interpretations (Giorgini et al. 2015a, b), or because they are hindered from following them (Resnik 2005).

By challenging CRE, the RRI concept constitutes a further step in the history of policy debate on how and in what ways science, technology and society should relate and engage with each other (Stilgoe and Guston, referred to in Felt (2017). So far, the governance of RRI is most frequently characterised by its four dimensions: *anticipation, reflection, inclusion* and *responsiveness*. These were first proposed in 2013 by van den Hoven (2013), and further developed by Stilgoe et al. (2013). Other policy keys have been suggested as the main constituents of RRI, these inlcude *ethics, social engagement, gender equality, open access* and *science education* (Geoghegan-Quinn 2012), as well as *justice* (Schroeder and Ladikas 2015), *knowledge management* (Lubberink et al. 2017), *care* and *sustainability* (Burget et al. 2017; Strand et al. 2015). So far, however, they have been unable to replace or supplement Hoven et al. and Stilgoe et al.’s ‘Famous Four’.

In line with the four dimensions, researchers practising RRI are tasked with anticipating and reflecting on the societal and environmental consequences of the R&I process, among other things by establishing interdisciplinary research teams and through the active involvement of stakeholders, preferably from the very beginning (e.g. (Owen et al. 2012; Van den Hove et al. 2012). The researchers are expected to be responsive to the anticipation and reflection processes, as well as to the project’s outcome and impacts. To what extent, and under what conditions, stakeholders can be expected to be co-responsible for the process’s consequences remains somewhat of an open question. The overall aim is to reduce the risk of contested innovations and create a more sustainable society.

Researchers’ responsibility to not cause harm to society or the environment has also been emphasised in literature on CRE, for example in Resnik (2005) textbook on research ethics; the Code of Conduct for Nanoscience and Nanotechnology Research (Commission of the European Communities 2008); guidelines for ethics in science and technology (e.g. (The National Committee for Research Ethics in Science and Technology 2008)); guidelines for social sciences, law and the humanities (NESH 2016); and the Declaration of Helsinki – Ethical Principles for Medical Research Involving Human Subjects (World Medical Association 2001). Researchers’ responsibility to not cause harm to society and the environment includes the health of humans and those directly affected by the research: people, animals, insects and plants. However, whereas societal and environmental concerns represent a key responsibility in RRI, the literature on CRE typically treats them as just some of the several types of concerns.

Another type of concern in CRE regards the researchers’ responsibility to do ‘proper research’, that is, to develop new, solid and verifiable knowledge and thereby ‘get (verify/falsify) proof of concept’. In essence, these concerns deal with methodological issues and aim to ensure the quality of the data material and analyses. As these concerns represent the *raison d’être* of science, they also form part of RRI practices, yet are often taken for granted and are less or indirectly articulated. In some RRI literature, the concern with conducting proper research has been expressed as a barrier to practising RRI. A telling example is a study conducted by van Hove and Wickson (2017, 227) concluding that some aspects of RRI are hard to conduct in practice because they challenge researchers’ perceptions of ‘good science’, characterised as ‘independent, objective, the domain of experts and most productive when left to its own device’.

In the literature on CRE, we also find a third type of concern relating to actors directly involved in the R&I process. These concerns, among other things, regard the impact of research on health, safety and the environment (HSE), the project partners’ career development, the recruitment of new scientists and the collaboration between project partners. Concerns relating to other actors are addressed in the RRI dimension ‘inclusion’. However, whereas CRE first and foremost stress the concerns relating to project partners, and, to some extent, research subjects, RRI practices emphasise establishing interdisciplinary research teams and including external stakeholders. The involvement of new actors with different ethical concerns has put conventional ways of organising the R&I process under pressure (Nathan 2015; Fossum et al. 2018).

A fourth type of concern regards contractual issues, here referring to the researchers’ responsibility to complete the R&I process within the schedule and budget, typically *vis-à-vis* research funders (Scriven and Coryn 2008). Both private and public funders of scientific research expect quicker and more tangible return on investments (van den Burg and Swierstra 2013). In contrast to the above-mentioned concerns, these forms of contractual concerns are not rooted in scientific ideals, but in economic liberal ideas that gained ground in the public sector in the 1980s, emphasising the importance of cost effectiveness, cost cutting and budget discipline (e.g. Schedler and Proeller (2000)). A basic idea is that public money really is the taxpayers’ money, and the more cost-effective the research is, the more money will be available for other worthy causes (Schroeder and Ladikas 2015). When these kinds of concerns have been discussed in the RRI literature, they have typically been referred to as contextual factors, frameworks, structural conditions etc. and not regarded as moral principles. However, as they involve a choice between completing the R&I process within the schedule and budget, or giving priority to other research principles, they do represent a potential ethical dilemma. In RRI, contractual concerns may not only challenge the responsibility of developing new, solid and verifiable science, but also the responsibility of achieving the socio-economic effects (jobs, wealth and well-being) on society that are often promised in research applications after having been encouraged, if not required, in research calls (Zwart et al. 2014).

From this discussion, two observations can be made: Firstly, there appear to be sources of dilemmas in and between the principles of the CRE and RRI dimensions with regards to the research’s *raison d’être*, the importance of societal and environmental concerns in R&I processes, affected parties and contractual issues. These sources of dilemmas are summed up in Table 1.

**Table 1:** Sources of dilemmas in and between conventional research ethics and Responsible Research and Innovation dimensions

Sources of ethical dilemma	Principles of CRE	RRI dimensions
The role of societal and environmental concerns	Social and environmental concerns are two of several ethical concerns	Social and environmental concerns should be given higher priority than other concerns
The research’s *raison d’être*	Falsify/verify proof of concepts	Invent and develop responsible and socially robust innovations
Affected parties	Concerns relating to project partners and other actors involved in the research processes, e.g. HSE, career development etc	Projects should be conducted by interdisciplinary consortia and include external stakeholders that could be affected by the research
Contractual issues	Conduct the project within the schedule and budget	Conduct the project within the schedule and budget, as well as fulfilling promises made about ‘effects’ (i.e. impact). This will pay off both during research, but even during commodification and commercialization

**Fig. 1:**
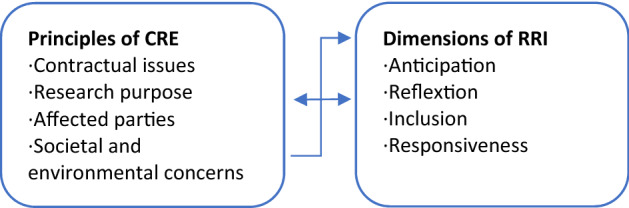
Principles of the Conventional Research Ethics and Responsible Research and Innovation dimensions

As illustrated here, the principles of CRE and the RRI dimensions address some of the same topics, yet in different ways. Whereas CRE focuses on ensuring the project partners’ interests during the R&I process, RRI focuses on including the relevant perceptions and competences of interdisciplinary project partners and stakeholders. Keeping to schedules and budgets is important in both CRE and RRI. Other concerns are prioritised differently in CRE. Most importantly, the societal and environmental concerns are given higher priority in RRI than in CRE. Society’s desires and concerns need to be responded to and taken into consideration (‘responsiveness’). The greater emphasis on social acceptance makes socially and environmental issues the key concerns of RRI. As illustrated in Fig. 1, societal and environmental issues are not one of many principles, rather a premise; *the* concerns around which all other dimensions revolve. Moreover, RRI is most likely not only influenced by CRE, but also influencing it, implying that CRE cannot be understood as a piling immovable in its social–historical stability, but is rather a subject of constant renegotiation in research ethical committees at national and international scale until it eventually—and not without opposition—is slightly updated in line with the contemporary zeitgeist (cf. Kuhn’s paradigm shifts).

As we will come back to in the discussion of this paper, Fig. 1 opens for two alternative interpretations of RRI as a concept: A visionary *broad approach*, where it is seen as a new way of doing science that replaces conventional research practices. From this perspective, the alignment of research and innovation to the values, needs, and expectations of society is expected to be achieved by giving higher priority to societal and environmental concerns than other concerns (Stahl et al. 2019); and a *narrow approach*, where RRI is seen as more of a supplement to conventional research practices. The alignment of science and society should be achieved by emphasising societal and environmental concerns more than is currently the case, yet not necessarily in a way that jeopardises other concerns (see e.g. Kuzma and Roberts 2018). Although both approaches are represented in current literature on RRI, our general impression is that the broad approach is more frequently represented by scholars who address RRI as a concept, whereas the narrow approach is more frequently represented in the smaller body of literature addressing RRI in practice.

From this outline, two implications for further analysis can be derived: Firstly, challenges and dilemmas experienced by researchers could be the result of different ethical concerns rooted in CRE and RRI. Secondly, the gap between RRI as a concept and as a practice may be the result of difficulties in practicing the broad approach to RRI. Due to individual and environmental barriers, a narrow approach to RRI is applied in practice. It is these potential dilemmas and barriers that will be further addressed in this paper.

#### Methodology

The analysis is based on a qualitatively oriented case study of the BioConcrete project (pseudonym). In qualitatively oriented case studies, the subjects of the study are typically approached from different angles by means of different sets of data. Although idiosyncratic in nature, we expect the research results to be ‘naturalistic generalisable’ (Ruddin 2006), that is, useful for others in terms of addressing a R&I process from which lessons can be learned. Moreover, although the research results cannot be generalised in a quantitative sense, they represent some of many observations on which generalisations can be made in the future (Flyvbjerg 2004). In this study, social scientists took part in the research process using critical reflection and research techniques as methodologies playing double role as ‘participants’ (or insiders) and ‘observers’ (or outsiders). As participants, they listened to the informants’ arguments without distinguishing between ‘right’ or ‘wrong’ (Kemmis and McTaggart 2000), and as observers, they critically reflected on, challenged and discussed these arguments in light of the RRI’s goals and recommended techniques for bringing science closer to society.

A description of the BioConcrete project is given in the Text Box.

**Table:** 

Text Box: The BioConcrete project
The analysis is based on observations made in the BioConcrete project conducted from 2014 to 2017. The project was funded by the IdeLab program of our National Research Council and aimed at developing a cement with significantly reduced energy demands and CO2 emissions compared to more conventional production methods. The basic concept of the BioConcrete process was to use bacteria to produce acid to partially dissolve calcium carbonate (CaCO3 or calcite) and, subsequently, induce an increase in pH to initiate calcite reprecipitation. The precipitated calcium carbonate would act as a binder between grains of sand and other aggregate materials
The project included seven work packages (WPs): (1) project management; (2) development of the process of cementation by in situ recrystallisation of calcium carbonate; (3) microbial strain and process development; (4) integration and optimisation of bio-induced recrystallisation; (5) life cycle assessment; (6) ethical challenges and solutions within the framework of the EU Code of Conduct; and (7) dissemination, communication and intellectual property management
The BioConcrete consortium comprised twelve internal stakeholders: microbiologists, chemists, biophysicist, engineers and social scientists, with a core of eight people. The microbiologists, chemists and biophysicist were responsible for product development. An engineer represented the industry based on his knowledge about the cement business-to-business market. Another engineer was responsible for the life-cycle analysis. The remaining two researchers were social scientists who were responsible for the project’s ethical discussions and concerns. In addition, the project included an advisory group of external stakeholders: one microbiologist, one chemist and one architect, who took part in one or two project meetings each

As shown here, the project was funded by the National Research Council’s IdeaLab programme. The program idea stemmed from a sandpit workshop methodology developed by the UK Engineering and Physical Sciences Research Council (Giles 2004). Given the strong emphasis on ethical and societal components in the IdeaLab programme, the BioConcrete project started out by taking more of an ELSA approach to these issues, but soon adopted the emerging RRI framework.

The core of the BioConcrete consortium consisted of twelve members, here referred to as *researchers*. In the analysis, the members were divided into two categories: *the scientists* (the microbiologists, chemists and physicists) and the *social scientists,* who, due to their roles in the project, included the engineers. The scientists were the main responsible for the project’s ‘research’, i.e. to make the concrete, whereas the social scientists were the main responsible for addressing the concrete’s societal and environmental impacts, including considerations of the concrete’s future market potential and make Life Cycle Assessment (cf. ‘the moral division of labour’ Rip and Shelley Egan 2010; Shelley Egan 2011; Rip 2017). The project was based on a linear innovation model, where the basic research was conducted from the very beginning, whereas the applied research tended to be conducted at later stages (Godin 2006).

Six sources of data from the project are used here: (1) Interviews with project partners, face to face or on Skype, individually or in groups (the interviews were taped); (2) Project meetings conducted face to face, twice a year, of which half of the meetings were conducted with members of the advisory board. The project meetings were conducted over two days, during which one hour was set aside for discussing ethical and societal issues; (3) Observations in laboratories combined with interviews (these were videotaped); (4) Observations and interactions in the project’s weekly online meetings; (5) Written text stored in the project’s Dropbox, such as literature suggestions, minutes from online meetings and material datasheets; and (6), a feature article, in which the project partners review and reflect on the lessons learnt during the BioConcrete, published in an online, scientific magazine in 2017.

The analysis was conducted by the social scientists and based on grounded theory and narrative methodology. Grounded theory uses inductive reasoning, in contrast to the hypothetic-deductive model of scientific method (Glaser and Strauss 1967). In narrative methodologies, researchers typically collect and review data, looking for repeated arguments or ‘stories’ that people use to make sense of their life (Mitchell and Egudo 2003). The research questions raised in this paper were formulated after the data was collected, when the social scientists discovered that most of the arguments addressed problems and dilemmas faced by the researchers when they tried to apply RRI for the first time. As these arguments were for the most part articulated during interviews and, to some extent, project meetings, the analysis is primarily based on dataset 1 and, to some extent, datasets 2 and 6.

The analysis was carried out in three steps focusing on (1) dilemmas between various principles of CRE (contractual, purpose, collegial and societal); (2) dilemmas between RRI dimensions (anticipation, reflexivity, inclusion and responsiveness); and (3) dilemmas between the principles of the CRE and RRI dimensions. In this paper, we will focus on the analysis conducted in the third step, but draw on observations made in the first and second steps where relevant.

The role of the three elements of practice (meaning, competences and materials) in these dilemmas were also considered in the analysis. As shown in the theoretical section, most research on RRI practices has hitherto addressed the elements of understandings (in terms of concepts and approaches) and of competences (in terms of information and power asymmetry), whereas the role of the material element has arguably received much less attention. Special consideration was therefore paid to the material element of research practices and how it relates to the understandings and competences elements.

## Results

In line with CRE, the scientists argued that they always tried to anticipate ethical challenges in their daily work, yet not necessarily explicitly.

I think that most researchers are responsible persons who try to consider the challenges anyway…We think about ethical questions in the same way as we are thinking about ethics in everyday life, at work, at home. We always have some internal discussions about what is right and wrong. (Scientist in an interview).

The problem was that it was hard to anticipate such matters at an early stage in the process since they did not know the project’s outcome (cf. the Collingridge information dilemma). The researchers were, for example, not sure if they would be able to make a workable bio-based cement at all.

The conventional goal of achieving proof of concept was, in this respect, given priority at the expense of the RRI dimensions of anticipating societal and environmental impacts from the very beginning of the R&I process. The postponement of the anticipation dimension was based on a socially constructed division between ‘research’ and ‘innovation’, i.e. basic and applied science (Gibbons 1999).

Science is different from innovation…Science is about making a hypothesis and testing whether it is true or not. This project is a science project at this stage. It is supposed to contribute to innovation, but first you have to test your hypothesis. We want to bring it towards the next step. We make one brick. How to make more sustainable bricks in a sustainable way is the next step; innovation. (Scientist in a project meeting).

The first step was, in other words, to test whether it was possible to make bio-based cement at all. If so, changes would be made to make the cement (more) sustainable. At this point in time, it would not make any sense to worry about the bio-cement’s potential social and ethical impacts:

We are still very far from the point where it is used, but when we get closer to that point, we should think about it. I am strongly against worrying about problems that may never be realised. The study of nanotechnology, for instance, is full of problems that are science fiction. (Scientist in an interview).

Another dilemma that can be associated with the Collingridge problem of control can be found in the researchers’ discussions about bacteria and their potential harm to society and the environment. The discussion started early in the project when the microbiologists considered using genetically modified (GMO) bacteria:

You don’t take bacteria from nature, manipulate them in the lab and put them back again in nature. That might disturb the balance in nature. (Scientist in a group interview).

The loss of control did also regard the potential scepticism that the project may trigger in the public:

“People’s perception of bacteria is different from ours. Ours is facts-based…People should not be scared of bacteria per se, but, of course, they still might be. (Scientist in a group interview.)

Due to this concern, the social scientists were asked to do a desktop research of previous research on people’s attitude towards bacteria in concrete, preferably in a national context. The research revealed that no such study had been published either nationally or internationally.

A third dilemma was connected to an asymmetry of information and competence between scientists and the social scientists. The social scientists were included in the consortium to assist in a dedicated effort to anticipate among other things, societal, ethical and environmental impacts. Bacteria are a crucial element to realize the research and eventual innovation. This set the microbiologists in a privileged position, as they have bacteria and other microorganisms as their primary research interest. This entails the implication that the social scientists could encourage the microbiologists to reflect on potential harm, but were unable to fully judge the validity of their arguments.

If I had been a microbiologist or an expert on cement, I would have understood what it meant to change from one bacteria to another, whether it is important for the environment or not. But in [the kind of research I do] you seldom possess these kinds of knowledge. (social scientist in an interview).

The researchers’ solution to this problem was that the microbiologists informed the social scientists about the project’s potential harm and came up with solutions to how these risks could be handled. The social scientists, on their part, trusted the microbiologists’ information and judgements. At stake was the project partners’ possibility of realising proof of concept within the scheduled time:

I trust the leader of the microbiologist team. They [the scientists] have control over the time they have spent and that we don’t go in the wrong direction. (social scientist in an interview).

The asymmetry between the scientists and the social scientists came also to expression in material (and digitalised) conditions, such as project plans and budgets, the researchers’ access to labs and lab instruments, as well as rooms for physical project meetings, which were always located at the scientist’ universities. That being said, many activities that took place during the BioConcrete project evened out some of the asymmetry. For example, all researchers took part in weekly virtual meetings and were invited to the project's physical project meetings arranged every six months. To increase the social scientists’ understanding of the scientists’ work, a simplified language was used, which, unintentionally, improved the understanding and communication between the microbiologists, the chemists, and the biophysicists as well (Røyne et al. 2017). At each physical meeting, one or two hours were reserved for ethical reflection facilitated by the social scientists. A fourth dilemma observed in the BioConcrete project was between contractual concerns on the one hand, and the concern with performing proper research on the other. If the RRI dimensions of anticipation and responsiveness were given priority, the researchers weighed this against the risk of not being able to do the research properly on time and on budget; a research principle they were trained to prioritise. If they were no longer expected to follow this goal, the work lost its meaning:

My interest is really in providing proof of concept…If we cannot prove that the concept is viable, there is no point in continuing the project. (Scientist in an interview).

The solution to the problem was to postpone the anticipation and responsiveness processes to later stages of the project. The postponing was not only legitimised by a concern related to the project partners and research funders, but also to society at large. If the scientists failed in getting proof of concept, a more sustainable cement may not be developed and public money that could have been spend on other good causes would have been wasted.

We [the researchers] are still paid by the taxpayers. We have to remember that. We don’t put our own money into this. It is money from the government. (Scientist in an interview).

A complicating aspect of implementing RRI in the BioConcrete project was that the researchers were not sure when anticipation, reflection, inclusion and responsiveness processes had taken place (cf. the problem of indefinite definitions). For example, could the anticipation of societal and environmental impacts be based on the researchers’ gut feelings, or should they be based on more profound analyses, such as reviews of previous research or new data collection and analyses? Indeed, there were examples of changes made in the project period that might have been acts of responsiveness (e.g., the desktop study of people’s attitude towards bacteria that was carried out after a discussion about the project’s potential harm to society), but in general, it was hard to tell whether changes were caused by RRI activities and not the result of other events.

A general observation from this research has been that the BioConcrete project seemed to take a ‘pragmatic approach’ to RRI dimensions. The dimension concerning the inclusion of interdisciplinary researchers and stakeholders, which was the only dimension explicitly required in the call text, had been complied with. The dimension of reflection was also practised. In fact, it was through ethical reflections conducted in interviews and project meetings that most of the problems and dilemmas identified in this research were ascertained and discussed. The dimensions of anticipation (beyond gut feeling) and responsiveness did, however, bring forth dilemmas and were therefore for the most part postponed to later stages of the project, or even beyond the project period, when they did not pose a risk of jeopardising the researchers’ goal of achieving proof of concept (within the schedule and budget).

## Discussion

Table 2 sums up the BioConcrete researchers’ problems and dilemmas and their connections to the principles of the CRE and RRI dimensions, as well as how these challenges were solved by the researchers and how they could alternatively have been solved.

**Table 2:** The BioConcrete researchers’ challenges, dilemmas, andsolutions

	1	2	3	4
Challenges	Problem of scarce resources (1)	The Collingridge problem of control (2)	Problem of asymmetric competences (3)	Elusive understanding of RRI (4)
Experienced dilemmas	Giving priority to realisation of proof of concept or to finalise the project within the scheduled time if the research could take more time than budgeted	Doing or not doing RRI if the social and ethical consequences cannot be anticipated at this stage anyway	Doing or not doing RRI if this will take attention away from the scientists’ work of realising proof of concept within the scheduled time	Applying or not applying the broad approach to RRI if a narrow approach is sufficient
The researchers’ solutions	Make a real effort to realise proof of concept throughout the project period	Postponing elements of RRI until more information has been obtained	Trust the scientists’ solution of postponing elements/dimensions of RRI	Applying a narrow approach to RRI

As shown Table 2, column 1, one of the BioConcrete researchers’ greatest concerns was to realise proof of concept within the scheduled time. If this realisation took more time than scheduled and budgeted, the researchers could either choose to halt and wrap-up the project before proof of concept had been realised; if possible, extend the deadline with pertaining monetary loss; or submit a follow-up study, with the risk of not getting funding. To avoid this dilemma, the scientists made a serious effort from the very beginning of the project to push for proof of concept as early as possible. Activities that might disturb this mission, such as conducting RRI activities that incorporate all its dimensions, were selectively reduced to just reflexivity, with a more limited interest in anticipation and responsiveness. The researchers’ selective way of using RRI (van Hove and Wickson 2017) cannot be viewed independently from the fact that the R&I project was planned as a linear process, with basic research activities at the beginning and more ‘market-oriented’ innovation activities at the end. It can also be associated with Collingridge’s (1980) problem of information and control (cf. Table 2, column 2). If the BioConcrete researchers implemented a broad conception of RRI, they feared that their chances of realising proof of concept could become slimmer. However, if they did not, the risk of causing societal and environmental harm could increase.

Also, in line with previous research (cf. da Silva et al. 2019; Felt 2014; Levidow and Neubauer 2014), an information asymmetry between the actors involved in the R&I process caused a dilemma in the BioConcrete project (Table 2, column 3). The social scientists were expected to anticipate the societal and environmental impact of the project, but since this work presupposed data and competence that only the scientists possessed, the social scientists’ anticipation process was strongly dependent on the scientists’ assistance. If the social scientists were to conduct the anticipation process, they would need assistance from the scientists who would thereby spend time on RRI that could have been spent on realising proof of concept. However, if the social scientists did not conduct the anticipation process, the innovation could be less desirable from a societal perspective.

The analysis also supports previous research indicating that an elusive understanding of RRI may cause a dilemma between doing and not doing RRI (Table 1, column 4). Theoretically, RRI has been interpreted and understood as a new way of doing science (e.g. Von Schomberg 2013; Stilgoe et al. 2013). However, as this ambition has not been clearly defined, the BioConcrete researchers were able to define their level of ambition themselves. If they applied a *narrow* approach to RRI, the chance of realising proof of concept may increase. However, if they applied a *broad* approach, the risk of causing societal and environmental harm may decrease. The researchers’ solution to this dilemma was to concentrate on the reflection and inclusion dimensions of RRI and to postpone the anticipation and responsiveness dimensions to later stages of the project period. A more concrete RRI concept that clearly affirmed the scope of RRI might have solved this problem.

Overall, the study confirms previous research indicating that there is a gap between RRI as a concept and in practice (Stilgoe et al. 2013; Stahl et al. 2013). At the research level, the gap can reflect a broad versus narrow approach to the RRI concept, where researchers tend to apply the latter due to other concerns rooted in CRE and their manifestations in project descriptions and budgets. At policy level, the gap can reflect different levels of ambition, where politicians in theory aim at large-scale change (based on a broad approach to the RRI concept), but have in practice facilitated small-scale change (based on a narrow approach to the RRI concept). What the different levels of ambition may imply and how they influence the change from conventional research practices to RRI practices are illustrated in Table 3.

**Table 3:** Narrow versus broad approaches in relation to Responsible Research and Innovation practices

	Narrow approach to RRI	Broad approach to RRI
Understandings	Increase scientists’ understanding of RRI as a supplement to CRE	Increase all researchers’ understanding of RRI as a main concern and make RRI mandatory throughout the research process
Competences	Increase scientists’ competence in practising RRI as a supplement to CRE so that the ‘need’ to outsource RRI to the SSH scholars diminishes	Restructure the educational system so that SSH scholars’ competence in natural sciences increases, and vice versa
Materials	Changes in scientists’ project plans and budgets, textbooks and tools	Change project plans and budgets, textbooks and tools, and ensure SSH scholar access to labs, instruments, meeting rooms etc. when this is deemed necessary

The *narrow* approach to RRI is usually carried out by the scientists themselves without assistance from researchers in the social sciences or humanities. RRI textbooks, courses and tools are developed and offered to scientists in order to increase their understandings of and competences in RRI.

A *broad* approach to RRI, on the other hand, will, at the most extreme, represent a new way of doing science, where societal and environmental concerns are given priority at the expense of realising proof of concept. If the research represents a risk of causing harm to society or the environment, actions will be taken to stop or change the direction of research.

## Conclusion

In this paper, we have explored problems and dilemmas faced by researchers when they are expected to apply mandatory RRI for the first time. We have also examined how the researchers solved these challenges, and how they alternatively could have been solved.

The research supports previous research indicating that researchers face different ethical problems and dilemmas when they are expected to implement and apply mandatory RRI for the first time, such as a difficulties with anticipating and controlling future impacts, an asymmetry of competence between partners, stakeholders and the general public, and an elusive understanding of the RRI concept. The challenges were rooted in different principles of CRE and RRI dimensions and could be boiled down to one core dilemma: If RRI was applied from the very beginning of the project period, the chance of realising proof of concept within the scheduled time may decrease. However, if RRI was not applied, the risk of causing societal and environmental harm may increase. The researchers’ solution to this dilemma was to prioritize proof of concept and apply a narrow approach to the RRI concept. i.e., focus on some of the RRI dimensions while postponing others to later stages of the project period.

The research thereby supports previous studies indicating that there seems to be a gap between how RRI is expected to be applied in theory (RRI as a *concept*), and how it is applied in real research settings (RRI as a *practice*). In most theories on RRI, a broad approach to RRI is used, in which RRI is seen as a new way of doing science that replaces conventional research practices. From this point of view, societal and environmental concerns should be given higher priority than the realisation of proof of concept. This research has, however, indicated that a narrow approach to RRI is applied in practice, where RRI is treated as if it were a supplement to conventional research practices. The gap between RRI as a concept and a practice can thereby reflect different approaches to and levels of ambition regarding RRI.

The fact that the main observations presented in this paper support previous studies indicates that the research results can to some extent be generalised to studies of similar R&I processes. Such generalisations are, however, hard to make since all R&I processes have, to some extent, their own unique challenges. More studies on similar R&I processes are therefore needed before generalisations can be made.

To understand the unavoidable idiosyncrasies of this study, the results should be compared with studies of different R&I processes. (1) This research has focused on a research consortium including scientists, engineers and social scientists, coordinated by a scientist. Would the problems and dilemmas have been different if the consortium had been composed in a different way? (2) This research has addressed an R&I process in which RRI was mandatory. What problems and dilemmas would have been encountered if the RRI had been initiated by the researchers themselves? Finally, (3), this research has focused on problems and dilemmas faced by researchers in the first year of the project period. Would other problems and dilemmas have been identified had they been explored at a later stage of the R&I process?

Overall, this research has shown that researchers’ problems and dilemmas when they are expected to apply RRI for the first time were highly influenced by (and influenced) the environment and institutions of which the research project formed part. Roughly speaking, in the choice between proof of concept or RRI, researchers chose the former. It has also indicated that the change from conventional research practices to RRI practices is not made in a day, as it alters deeply founded cultural ideas about what science is and the competences and materials needed to do it. Amongst others, a baseline understanding of what it means to apply RRI must be clarified and RRI activities must be mandatory and budgeted throughout the research process. Overall, the educational system needs to be restructured so that project partners, stakeholders and the public can be more egalitarian in the future. As such pervasive changes cannot be up to individual researchers or consortiums alone but need the engagement of those with the power to change the environmental conditions impeding change, more effort is needed at the political level. The need for change has also been stated in other studies addressing research practices, for example in the RRI practice project, which recently launched a policy brief arguing for the redefinition of scientific excellence to include broader societal concerns (Owen et al. 2019). If changes at the structural level are realised, some of the limitations encountered at individual or group level might be easier to overcome.

## Data Availability

The data are not available.
